# Effect of a Simple Versus a Complex Matrix on the Polarity of Cardiomyocytes in Culture

**Published:** 2006-03-30

**Authors:** Rachel A. Davis, W. Barry van Winkle, L. Maximilian Buja, Brian J. Poindexter, Roger J. Bick

**Affiliations:** Department of Pathology and Laboratory Medicine, University of Texas Medical School at Houston

## Abstract

**Objective:** The objective of this study was to observe the effects of cell culture on cellular polarity in cardiomyocytes as influenced by cytoskeletal proteins. **Methods:** Cardiomyocytes from adult and neonatal rats were isolated and grown on 2 different extracellular matrices—laminin and a complex, fibroblast-derived extracellular matrix, cardiogel. The location of a number of proteins was visualized by means of fluorescence deconvolution microscopy, using specific fluorescent probes for α-adrenergic receptors, β-adrenergic receptors, the sarcolemmal L-type calcium channel, and the sodium + potassium adenosine triphosphatase pump protein. Intracellular migration of these proteins during the first 4 days of culture was followed and microscopic stacked images were reconstructed. A fluorescein isothicyanate–labeled probe for actin was used to ensure that cardiomyocytes were being examined, based on protein patterns. **Results:** We examined 2 types of myocyte: freshly isolated neonates and cultured adult cardiomyocytes that undergo dedifferentiation. Initial, perinuclear clumping (endoplasmic reticulum/Golgi-associated) of the probes with an ensuing spread to the cytoplasm and periphery, accompanied by a better organization and more rapid response to biochemical stimuli, was seen on the complex matrix. **Conclusions:** A complex matrix overcomes cell polarity at a faster rate than myocytes cultured on a simple matrix, although both culture matrices were able to support cell growth and differentiation, and single-layer cultures are a good method by which structural and biochemical data can be obtained. The use of a native, complex matrix is preferable to employing a simple, single protein, although temporal aspects of cell growth must be considered regarding the particular aspect of the cell structure development/biochemical pathways that the researcher intends focusing on.

Despite important developments in treatment, cardiovascular disease continues to be the principal cause of morbidity and mortality in the United States. The overwhelming impact of cardiovascular disease on the population has fueled significant research concerning cardiovascular function, of which an important aspect is the use of cultured cardiomyocytes. Progress in cardiac research by means of cell culture is determined by whether this method sufficiently resembles the cardiomyocyte in its natural physiologic state, while being a major tool for initial investigations as to whether cardiomyocyte implantation in areas of cardiac ischemia and fibrosis is a realistic and viable method for future healing and replacement therapies in the treatment of heart disease.

Interest in cell replacement and repair therapies for cardiac disease came to the forefront in the 1990s. An early study in a rat model^1^ suggested that the implantation of neonatal cardiomyocytes into areas of cardiac infarction was indeed a valid potential clinical therapy procedure to pursue. Further studies then shed some doubt on ischemic reversal^2^ by implanted myocytes, and brought us to the era of stem cells in tissue formation and regeneration.^3^,^4^ Within all these new proposals, there seems to have been a loss of recognizing that communication between a cell and its environment dictates biological processes such as development, inflammation, and immune responsiveness and that the adhesive characteristics of cells to their surroundings and extracellular matrix (ECM) are also very important in the regulation of cell function and tissue development,^5^ and that studies on cultured cells, and harvesting of cultured cells for implantation, needs to be considered in light of the recipient ECM.

Cell attachment to the ECM is predominantly carried out by integrins, a highly expressed family of transmembrane cell surface adhesion receptors. In addition to connecting individual cells, integrins transduce signals that manage cell growth, migration, and differentiation. Integrins cluster to form focal adhesions—these specific sites of attachment not only affording structural linkages between the internal actin cytoskeleton and the ECM, but also acting as a central site of signal transduction that directs cellular activity.^6^ Since the environment with which the cells interact plays a vital role in the organization and function of the cell, techniques for culturing are crucial regarding the properties of the developing cells^7^ and their potential therapeutic use. In the context of heart disease, it was reported that the ECM molecule tenascin-C not only was very important in heart tissue remodeling, but was an indicator of myocardial disease activity,^8^ again indicating the importance of the ECM composition.

Currently, monolayer culture is the common method of growing cells, involving the attachment of cells to a particular substrate, such as a petri dish or ECM component. Primary culture of cardiomyocytes involves the use of a variety of ECM components, in both simple and complex mixtures such as simple matrices like laminin, collagen, and fibronectin. Although often being used in cell culture, these simple proteins do not resemble in vivo cardiac ECM components sufficiently.^9^ A more complex matrix, one that closely approximates the ECM from a particular tissue of origin, is preferable. One such example is cardiogel, a fibroblast-derived ECM.^10^

In their native environment, cardiomyocytes exist in a 3-dimensional system, embedded in and linked together by a complex ECM.^11^ When cardiomyocytes are cultured on an ECM component, the myocytes will spread and undergo morphological transformations into a more 2-dimensional form,^12^ but under normal physiologic conditions we would expect to find receptor proteins and ion channels surrounding the cell, just as the ECM completely encircles the cell. However, since these receptors and channel proteins are normally in contact with the ECM we hypothesized that they would demonstrate a migration of proteins toward the monolayer employed in the culture, leading to cellular polarity. If this polarity does occur, understanding the changes in functional and metabolic responses could be valuable in furthering cardiovascular research, and something of importance to consider when culturing cells for implantation experiments.

## METHODS

### Experimental model

Neonatal cardiac myocytes were isolated as previously reported.^5^ Briefly, 2- to 3-day-old rats were sacrificed, the hearts removed and ventricles harvested. The tissue was digested with collagenase/pancreatin, separated on a Percoll gradient (Pharmacia, Piscataway, NJ) and cultured on laminin- or cardiogel-coated coverslips in Dulbecco's Modified Eagles Medium (DMEM; Gibco, Grand Island, NY) containing 5% HyClone calf serum (Logan, UT) and 5,000 U/L each of streptomycin and penicillin. Cultures were maintained in an atmosphere of 90:10 oxygen/CO_2_ at 37°C.

Adult myocytes were harvested from adult rats as previously described.^13^ Anesthetized rats (200 g average weight) were given 3000 IU of heparin by intravenous injection via the inferior vena cava. The heart was subjected to enzymatic digestion on a Langendorf ap tus, and myocytes were harvested by centrifugation.

For differentiation of the myocytes, 1 to 2 drops of the suspension obtained above was layered onto sterile laminin- or cardiogel-coated coverslips, and incubated for 30 minutes at 37°C in 5:95 CO_2_/air. Plating medium containing 10% fetal calf serum, 1:360 Ara-C, 1:1000 insulin, and 5000 U/L of streptomycin and penicillin was gently added and the coverslips were incubated as above and fed with fresh plating medium every other day.

### Isolation of cardiogel complex matrix

The fibroblast-derived matrix was isolated as described,^14^ using the mixed cell suspension for the myocyte preparation. The band at the density of 1.05 to 1.062 on the Percoll gradient is used for fibroblast purification. The cells are plated on plastic, incubated in 90:10 air/CO_2_, and grown to confluency over 3 to 4 days. The plates are treated with 0.5 mM EDTA solution (0.05% trypsin/0.5 mM Na_2_EDTA) to detach cells, and the remaining cardiogel matrix is washed in DMEM and used for the direct culturing of cardiomyocytes, either in the petri dishes or on 10-mm glass coverslips coated with cardiogel.

### Fluorescence deconvolution microscopy

Probes and antibodies were used to image and reconstruct cells to show adrenoreceptors and various proteins in cardiomyocyte cultures. We used DAPI for the nuclei, Texas Red and FITC tagged secondary antibodies for actin, tagged Prazosin for α-receptors, CGP-12177 for β-receptors, and Bodipy Green and Texas Red–labeled secondary antibodies for the sodium + potassium adenosine triphosphatase (Na+K-ATPase) and L-type calcium channel (Molecular Probes, Eugene, OR). Cells were reacted with the probes for 15 minutes at room temperature, placed under 1 drop of antifade (Elvanol, Dupont, Wilmington, DE), and visualized on an Olympus IX70 inverted microscope and scanned with an Applied Precision DeltaVision scanning deconvolution fluorescence microscope (Issaquah, WA). Stacked image reconstructions were made and image analysis was performed on a Silicon graphics workstation using SoftWoRx software (Applied Precision).

### Real-time spectrophotometry

We used the measurement of intramyocytic calcium to demonstrate biochemical parameters in parallel with the development of the cells and the migration of particular receptors and proteins. Myocytes, both cultured neonatal and adults, were treated with isoproterenol (10 μm) or ouabain (5 μm), and the Ca_*i*_ levels were measured with FLUO-3 fluorescence intensities using a Perkin-Elmer Concord System (Gaithersburg, MD), incorporating a SprectaMaster multiwavelength controller and temperature-controlled stage, equipped with a Rainbow Multi-wavelength Filter Wheel (Olympus America, Melville, NY). Images were acquired with an Olympix Astrocam CCD 4100 Fast Scan (12-bit, 768 × 576) camera at a rate of 1000 frames per second and 9 μm resolution as raw images. Contractile waveforms were reported from fast-caching acquisitions of 100 fluorescence intensities.

## RESULTS

We used a number of receptors and channel proteins to delineate the development of neonatal cardiomyocytes and to follow the dedifferentiation of isolated adult myocytes, comparing the influence of a complex versus a simple matrix on cell polarity.

**Figure 1:**
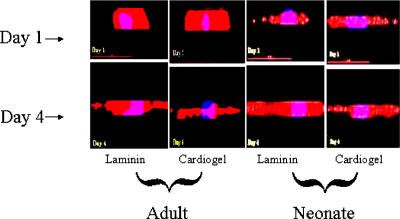
**Stacked image acquisitions of adult (4 left panels) and neonatal (4 right panels) cardiomyocytes cultured on laminin and cardiogel after 1 and 4 days in culture**. The top of the images is actually the coverslip/bottom of the cell. Red indicates the probe for the α-adrenoreceptors (gain boosted for clarity), while blue/purple is DAPI (nucleus). Magnification is × 600.

Figure 1 shows images obtained from deconvolution microscopic scans of a probe specific for α-1 receptors. Each panel demonstrates distribution on day 1 (upper images) versus that on day 4 (lower images) in culture.

The first 2 pairs are with dedifferentiated cultured adult cardiomyocytes, while the second 2 pairs are images obtained from neonatal cells. The first panel of each pair is with a simple laminin matrix, the second panel of each pair is with the complex, fibroblast-derived cardiogel. As one can see, the early (day 1) distribution of the receptor across and throughout the cell is greatest in the neonatal myocytes (right 2 panels), although little or no difference is seen in the distribution pattern when laminin-supported cells are compared to myocytes grown on cardiogel at day 4, where there is rapid migration of the receptors in the dedifferentiated cardiomyocytes, and the spreading of the probe is much greater in the cardiogel-supported cardiac muscle cells. Magnification is constant at × 600 and unit bars are 30 μm.

**Figure 2:**
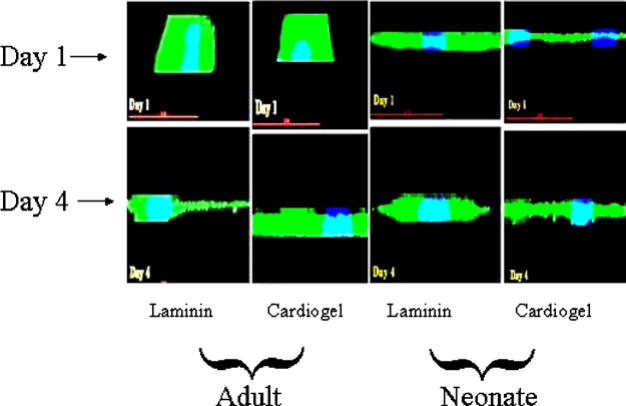
**Stacked image acquisitions in which we probed for the Na+K-ATPase pump protein. Top 4 panels are after 1 day in culture, bottom 4 panels are after 4 days in culture**. Green denotes the protein, and blue the nucleus. Magnification is × 600.

Figure 2 demonstrates a similar sequence of images, although this time we have targeted the Na+K-ATPase pump protein. Again, a similar pattern is seen, with the neonatal cells exhibiting an earlier diffuse pattern that does not change a great deal during 4 days in culture, while the dedifferentiated cells show a central localization of the probe on day 1, spreading by day 4, and greater spreading on the complex matrix.

**Figure 3:**
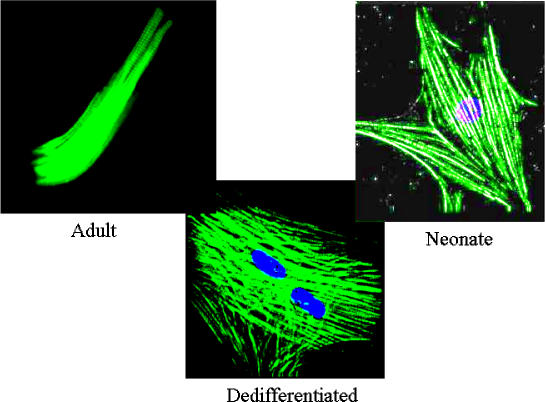
**The 3 cell types that were used for this study are demonstrated here, DAPI being used for the nucleus and FITC-tagged secondary antibodies for the cardiac actin**. This figure denotes the transformation of the elongate, freshly isolated adult cardiomyocyte (first panel); the large, stellate dedifferentiated cultured myocyte (middle, lower panel); and the typical, also stellate, neonatal myocyte (right panel).

Figure 3 shows the 3 types of cardiomyocyte employed in this study with (from left to right) freshly isolated adult, dedifferentiated cultured adult, and cultured neonatal cardiomyocytes. The green is actin, visualized with an FITC-tagged, cardiac-specific secondary antibody; the blue areas are nuclei, stained with DAPI (Molecular Probes, Eugene, OR).

**Figure 4:**
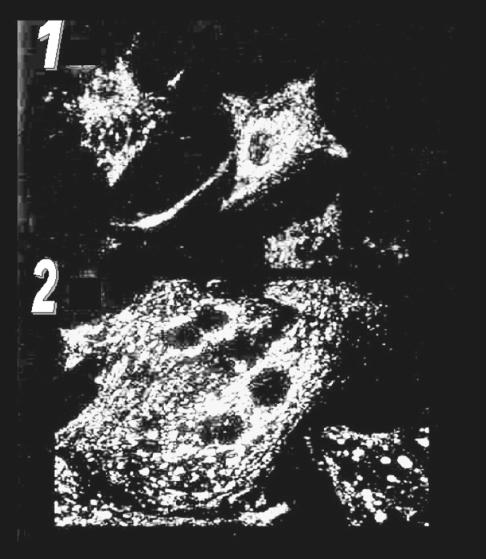
**Taken from one of our previous publications, the figure demonstrates the rapid development of intracellular organelles on the complex matrix (panel 2), compared to the simple matrix (panel 1)**. Here we have shown mitochondria probed with MitoTracker. Magnification is × 500.

Figure 4 is included from one of our previous publications^10^ and demonstrates that the complex matrix does lead to larger cells and the appearance of more subcellular organelles at an early stage. This particular pair of images shows neonatal cardiomyocytes that have been cultured on laminin (panel 1) and cardiogel (panel 2) for 4 days and then probed with MitoTracker (Molecular Probes) to reveal mitochondrial content.

**Figure 5:**
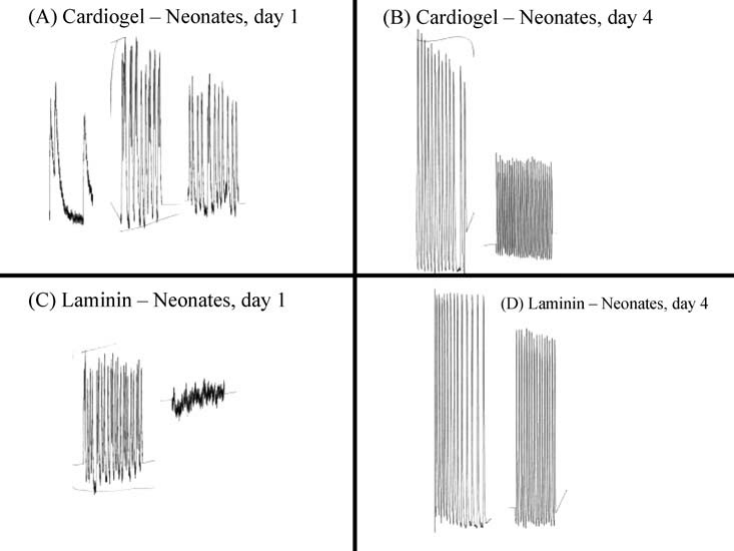
**Calcium transients following isoproterenol treatment, acquired by real-time fluorescence spectroscopy (as described under Methods)**. Note the initial lack of response on the complex matrix (panel A) but the rapid, smaller transients in panel B. Compare this with the lower panels, acquired on the simple matrix, in which there was a rapid, large response to isoproterenol treatment (panel C), but a much lower response after continued culturing (panel D).

Figure 5 is a compilation of transients acquired by real-time fluorescence spectrophotometry, detailing isoproterenol-stimulated calcium changes via the β-receptor/adenylate cyclase pathway. Comparing neonatal cells cultured on a complex (A) and a simple matrix (C), it is obvious that the effects of isoproterenol stimulation are quite different. The traces are a compilation over a 5-minute recording period, and show that on cardiogel there was somewhat sporadic beating, which was regulated and increased by isoproterenol administration, a pattern that remained regular although peak calcium was slightly reduced. However, in the cells cultured on laminin, the initial regular pattern of transients was blocked after an extended exposure of the myocytes to isoproterenol, and the intracellular calcium level remained high, as ascertained by the increased distance of the base of the transients from the baseline. By day 4, cultures on both matrices responded to isoproterenol stimulation, although the stimulated myocytes on cardiogel tended to have a slightly faster rate and a correspondingly faster cycling of calcium, as evidenced by the smaller peak height.

**Figure 6:**
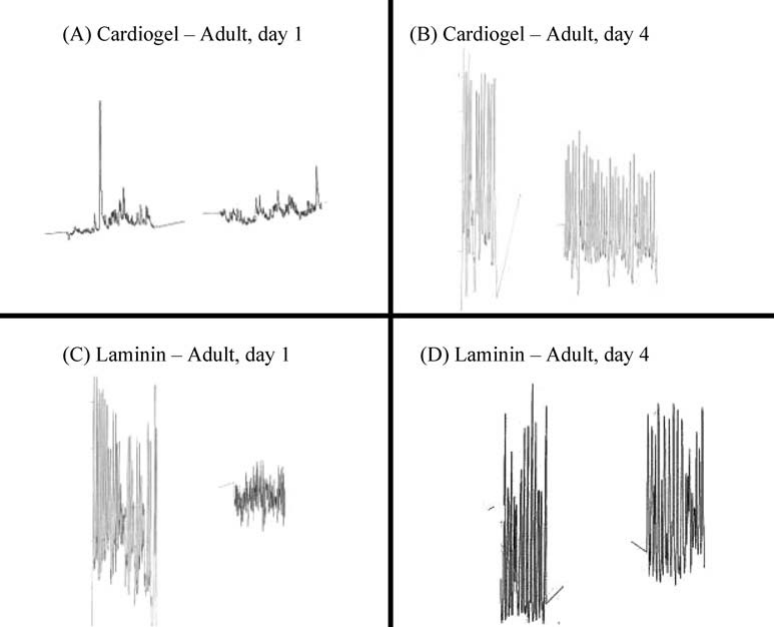
**A similar pattern to myocyte development was seen when ouabain was included in the medium, this time demonstrated with cultured adult cells**. Panels A and B, day 1 and day 4, respectively, demonstrate the eventual classic response to ouabain, with reduced calcium transients, but little or no effect on intracellular calcium levels (panel B). However, on the simple matrix, there is a rapid, complete blockade of calcium transients on day 1 (panel C), with a reduced effect after 4 days in culture (panel D), demonstrating the different effects culture matrices have on this particular pump protein.

Figure 6 is a 4-panel presentation comparing transients from cultured adult cardiomyocytes after treatment with 10 μM ouabain (Sigma Chemical, St Louis, MO). The myocytes cultured on cardiogel again revealed sporadic beating on day 1, and therefore an expected negligible effect following ouabain addition. By day 4, however, transients were regular and much larger, with ouabain producing a reduction in peak size and a more rapid rate, as would be expected. These findings contrasted sharply with transients acquired from cardiomyocytes cultured on laminin. Large, irregular transients were obtained on day 1, and treatment with ouabain caused an 80% reduction in transient height and increased intracellular calcium levels as ascertained by a raised basal level. By day 4, although the increased intracellular calcium levels at the end of each transient was still evident, the reduction in peak height was minimal.

## DISCUSSION

Myocardial dedifferentiation has been recognized as part of the group of repair and compensatory mechanisms that are initiated in cardiac disease.^14^–^16^ Many researchers currently use adult myocyte culture models to examine these changes and myocyte return to a natal or perinatal form.^17^,^18^ It therefore behooves us to determine if cultured cells do indeed reflect in vivo environments and that signaling pathways and calcium flux measurements in these models are useful in the understanding of cardiac cell function and heart disease, particularly when cultured cell harvesting is becoming more and more common for tissue regeneration and organ repair.

We used a number of markers to delineate the development of neonatal cardiomyocytes and investigate the dedifferentiation of isolated adult myocytes. We also examined the effects of both a simple and a complex culture matrix on cell growth, our initial hypothesis being that polarity of the cultured cells might lead to acquired data that is not entirely reflective of in vivo mechanisms. We also measured activities of 2 outer-membrane ion-exchange mechanisms, the β-adrenergic receptor via isoproterenol-stimulated intracellular calcium increases, and the Na+K-ATPase, via ouabain inhibition of compensatory intracellular calcium changes, to determine at what stage, if at all, the particular cardiomyocyte culture matrix influenced results from such experiments.

The general trend in the myocytes was for the complex, fibroblast-synthesized cardiogel matrix to enhance the distribution of the proteins throughout the cell and on the cell surface at a faster rate than found in myocytes cultured on the simple, laminin surface. However, this trend was not the same for all of the proteins probed, particularly as found with ouabain-inhibitable transients (Fig 6). Our theory regarding these protein distributions is that neonatal myocytes are actually in the process of forming the proteins in the nucleus-Golgi-endoplasmic reticulum complex, with the products then being distributed throughout the cell, often revealing a centralized, clumped pattern in early development. However, as can be seen, this centralized pattern was even more pronounced in early cultures of adult myocytes, probably due to a re-formation of proteins already present, and then a rapid, complex-matrix supported, distribution to appropriate intracellular locations, an undertaking that was usually complete by day 4 in the adult cultures, but slower in neonatal myocytes. Therefore, the cell polarity that does exist is overcome more rapidly culturing on a complex matrix.

Our conclusions are therefore that culturing of cardiomyocytes on a complex matrix enhances a rapid development of the cells in all aspects, that is, size, subcellular organelle development, proteins, etc, and also results in a quicker loss of cell polarity, possibly benefiting researchers in terms of interpretation of their results. Culture matrices and culture times therefore require very careful consideration, and it could well be an important point to consider when harvesting cells for implantation.

Previous work in our lab has investigated myocyte dedifferentiation and the possible consequences of this phenomenon in heart dysfunction,^13^ focusing on disruptions in signal generation and calcium fluxes. This current work demonstrated that as dedifferentiation occurs, there is a migration of proteins toward a central location, then redistribution, hinting at an attempt by the cardiomyocyte to form an early stage of the cell, possibly targeted at new cell formation. This theory receives some support from the fact that many binucleate myocytes are found in isolated adult cultures. These findings are therefore important in research directed at myocyte replacement strategies to overcome cardiac fibrosis and necrosis in situ, in which it might be important to culture fetal and neonatal cells to a point that they are fully functional and in such a manner that polarity is overcome and donor cells are ready to immediately interact with the ECM of the recipient tissue or organ.

Promise in cardiac cell transplantation to improve heart function has been shown with a number of cell types, including smooth muscle (19) and skeletal myoblasts (20). However a recent report (21) detailed poor viability of the transplanted cells, with death of the donor cells being rapid. These authors also noted that inflammatory response suppression also is desirable and that the cells are preconditioned. Perhaps, allowing the cells to develop to a later stage than is usually considered, and culturing these cells in such a manner as to allow polarity to be overcome, might be beneficial in both immediate activation and cell viability.

## References

[1] Scorsin M, Marotte F, Sabri A (1996). Can grafted cardiomyocytes colonize peri-infarct myocardial areas?. Circulation.

[2] Sakakibara Y, Tambara K, Lu F (2002). Cardiomyocytes transplantation does not reverse cardiac remodeling in rats with chronic myocardial infarction. Ann Thorac Surg.

[3] Smits AM, van Vliet P, Kassink RJ (2005). The role of stem cells in cardiac regeneration. J Cell Moll Med.

[4] Rezai N, Walinski H, Kerjner A (2005). Methods for examining stem cells in post-ischemic and transplanted hearts. Methods Mol Med.

[5] Kleinman H, Luckenbill-Edds L, Bannon F, Sephel G (1987). Use of extracellular matrix components for cell culture. Anal Biochem.

[6] Whittard JD, Akiyama SK (2001). Activation of B_1_ integrins induces cell-cell adhesion. Exp Cell Res.

[7] Juliano RL, Haskill S (1993). Signal transduction from the extracellular matrix. J Cell Biol.

[8] Imanaka-Yoshida K, Hiroe M, Yoshida T (2004). Interaction between cell and extracellular matrix in heart disease: multiple roles of tenascin-C in tissue remodeling. Histol Histopathol.

[9] Piper HM, Isenburg G, Piper HM, Isenberg G (1989). Techniques for isolation and culture of adult cardiomyocytes. Isolated Adult Cardiomyocytes.

[10] Bick RJ, Snuggs MB, Poindexter BJ (1998). Physical, contractile and calcium handling properties of neonatal cardiac myocytes cultured on different matrices. Cell Adhes Comm.

[11] Terracio L, Borg TK, Clark WA, Decker RS, Borg TK (1988). Immunohistochemical characterization of isolated and cultured cardiac myocytes. Biology of Isolated Adult Cardiac Myocytes.

[12] Severs NJ, Shovel KS, Slade AM (1989). Fate of gap junctions in isolated adult mammalian cardiomyocytes. Circ Res.

[13] Poindexter BJ, Smith JR, Buja LM (2001). Calcium signaling mechanisms in dedifferentiated cardiac myocytes: comparison with neonatal and adult cardiomyocytes. Cell Calcium.

[14] Holtz J (1993). Myocardial hypertrophy after myocardial infarct: what is the significance of phenotype changes in cardiomyocytes?. Herz Cardiovasc Dis.

[15] Lerch R, Tardy-Cantalupi I, Papageorgiou I (1997). Cellular recovery after ischemia: physiopathologic aspects. Arch Mal Coeur Vaiss.

[16] Guinamard R, Rahmati M, Lenfant J (2002). Characterization of a Ca2+-activated nonselective cation channel during dedifferentiation of cultured rat ventricular myocytes. J Memb Biol.

[17] LoRusso SM, Rhee D, Sanger JM (1997). Premyofibrils in spreading adult cardiomyocytes in tissue culture: evidence for reexpression of the embryonic program for myofibrillogenesis in adult cells. Cell Motil Cytoskeleton.

[18] Bird SD, Doevendans PA, van Rooijen MA (2003). The human adult cardiomyocyte phenotype. Cardiovasc Res.

[19] Yasuda T, Weisel RD, Kiani C (2005). Quantitative analysis of survival or transplanted smooth muscle cells with real-time polymerase chain reaction. J Thorac Cardiovasc Surg.

[20] Baj A, Bettaccini AA, Casalone R (2005). Culture of skeletal myoblasts from human donors aged over 40 years: dynamics of cell growth and expression of differentiation markers. J Transl Med.

[21] Burdzinska A, Berwid S, Orzechowski A (2005). Muscle cell transplantation: the ups and downs. Postepy Hig Med Dosw [serial online].

